# A Rebalancing of Financial Valuations and Expectations Moving Forward in the Telehealth Sector as the United States Moves Toward a Post-COVID-19 Reality

**DOI:** 10.2196/35857

**Published:** 2023-07-31

**Authors:** Nicholas Bettencourt, Conor John Wilson, Philippa Jaye Johnson, Fabian D'Souza

**Affiliations:** 1 Boston Strategic Partners Boston, MA United States

**Keywords:** telehealth, telemedicine, remote consultation, eHealth, internet, mHealth, mobile health, digital health, delivery of health care, telecommunication, web-based care, web-based medicine, customer, economy, economics

## Abstract

The telehealth sector of health care delivery experienced significant growth at the start of the pandemic as web-based care quickly became essential for the ongoing safety of patients and health care providers, such as clinicians and other health care professionals. After vaccines were introduced, however, telehealth companies lost value as the need for web-based care appeared to lessen. Presently, both existing telehealth companies and new entrants to the space are seeking ways to innovate, gain investor and customer buy-in, and overcome competitors. New companies are hoping to be seen not as pandemic-era substitutes, but instead as reinforcements to in-person care, valuable in their own right thanks to the convenience and technological advancements they bring. This struggle to reframe the value proposition, or perceived benefit, of telehealth is reflected in fluctuating stock prices and dropping valuations. This viewpoint summarizes the market volatility seen in the telehealth sector since the start of the COVID-19 pandemic and suggests potential opportunities for growth in the space. This is accomplished through a qualitative secondary research approach, leveraging contemporary sources, financial references such as Yahoo! Finance, and peer-reviewed literature to support predictions for the future market. We found that, in 2020, the size of the US telehealth market rose to US $17.9 billion and is estimated to reach US $140.7 billion by 2030. Additionally, digital health venture funding nearly doubled in 2020 over the prior 2 years with total funding rising to US $14.1 billion. However, these factors produced an oversaturated market in which the volume of supply was higher than demand, resulting in a sharp drop in valuations for some as vaccination rates climbed in 2021. In the face of this rebalancing, or return to normal following excessively high or unsustainable valuations, we suggest a possible path forward for telehealth companies in the postpandemic era. Suppliers’ current role in the telehealth space—whether health care industry incumbents, that is, traditional health care delivery systems and companies, or “telehealth-first” challengers—are especially relevant to the specific growth strategies they should pursue. Furthermore, consideration of the areas of medicine and characteristics that best lend themselves to web-based care may lead to a greater chance for long-term success in a postpandemic health care delivery system. In the future, we believe investors should expect a bullish market, that is, one characterized by growing share prices. Success is likely to occur in part through changing the actual models of care, as opposed to moving traditional care to a web-based format. The oversaturated market will likely condense into select established telehealth giants who were able to adapt to the changing landscape. While investors may be reasonably hesitant regarding individual telehealth companies, the industry can expect slowed but continued growth.

## Introduction

### Background

Amidst the chaos of the COVID-19 pandemic, a previously undervalued industry sprung to the forefront of investors’ consciousness. Telehealth companies, which facilitate the delivery of health-related services and information using telecommunication and electronic transmission, experienced significant growth at the start of the pandemic as patients and health care providers—such as clinicians and health care professionals—sought care options that limited their exposure to those around them. As work and school quickly became web-based in March 2020, so did health care. While COVID-19 changed every aspect of day-to-day life, the US health care sector was particularly affected, having been spared by previous outbreaks of highly transmissible infectious diseases like SARS [1], which, had they reached the proportions COVID-19 eventually did, may have resulted in both earlier preparedness and overall growth in the telehealth industry.

The rapid adoption of telehealth as the primary means by which clinicians in the United States delivered care across most medical specialties fueled investor enthusiasm and sent capital into a market that had hitherto witnessed only modest interest. Yet, it was unclear to what extent the uptake of telehealth would be sustained following the arrival of measures to mitigate the necessity to remain socially distanced, most notably the vaccines. When, in May 2021, eligibility restrictions on the SARS-CoV-2 vaccines were lifted, investors grew more cautious in their approach to telehealth businesses, as demonstrated by the gradual downward trend in average stock price across major industry players during the period [2,3]. Conceivably, this caution was fueled by fears that the appeal of telehealth to clinicians and patients alike would last only as long as in-person care could not be offered safely.

As we will discuss in this viewpoint, such fears, while warranted for some specialties, have not been completely realized; the pandemic revealed to clinicians and consumers that telehealth has merits beyond its capacity to limit the spread of COVID-19, and in specific situations, can successfully complement—if not entirely supplant—in-person care. Still, in the absence of another public health emergency for which widespread social isolation becomes necessary, telehealth is unlikely to see levels of use like those it did in the first months of the pandemic. This reality appears to have chastened investors and led to a rebalancing of the market. This rebalancing is characterized by a return to more modest, though higher than prepandemic, values of many of these stocks, suggesting the high valuations seen at the beginning of the pandemic were unsustainable or outside of the norm. Here, we discuss potential opportunities for the future use of telehealth, by distinguishing those areas in which telehealth has the greatest growth potential from those in which it will struggle to gain a foothold.

Informed by qualitative secondary research, we cover the rapid growth and fluctuation in publicly held telehealth companies throughout the COVID-19 pandemic and observe which factors most strongly predict continued success following the conclusion of the public health emergency. Our viewpoint draws on sources that were accessed between August 2021 and May 2023. Literature was retrieved from various sources, including databases such as PubMed and Google Scholar, and contemporary publications such as Bloomberg and McKinsey. Reuters Stock data were obtained from Yahoo! Finance and Refinitiv. Effort was taken to include a wide variety of educational sources and varying opinions to solidify any hypotheses.

Our viewpoint attempts to review the volatility of the telehealth market for a nonfinancial audience, and explain how these patterns, and the relative benefits and challenges to telehealth use, can predict the utility of this industry moving forward. Our review of these sources suggests that the strongest telehealth businesses will eventually stabilize and achieve continued growth by adapting to customer needs and innovating within the sector. Ultimately, there is cause for optimism and the long-term outlook on telehealth stocks should be bullish, especially for those companies best positioned to capitalize on the benefits these technologies provide over in-person care.

### Terminology

For the purposes of this research, we defined “telehealth” as the delivery of any health care service by a health care professional via electronic means to a patient in a different physical location, irrespective of the specific technology used. Examples and references used in the Viewpoint reference telehealth as delivered by both video calls, audio-only calls, and remote patient monitoring. To avoid ambiguity, we did not use the term “telemedicine,” except when quoting from sources in which it was used synonymously with telehealth.

## Telehealth: a Market Rebalancing

### Pandemic Growth

Telehealth was present in the US market prior to the spring of 2020. However, the pandemic allowed the sector to achieve accelerated growth. In 2019, the US telehealth market was valued at an estimated US $11.23 billion; by 2020, this had increased to US $17.9 billion, representing a compound annual growth rate (CAGR) of 59.4% [4,5]. Additionally, in 2018 and 2019, digital health venture funding was relatively stable with totals of US $8.2 billion and US $7.4 billion, respectively, but this amount nearly doubled in 2020 when total funding rose to US $14.1 billion [6]. The sudden visibility of telehealth to investors drove both an increase in funding and substantial fluctuations for publicly traded companies.

Yet, the heady days of bullish enthusiasm for telehealth seem to have passed, as evidenced by the drop in stock prices for telehealth companies. The telehealth market experienced substantial declines in valuation once COVID-19 vaccines were introduced in the fourth quarter of 2020 and infection rates began to drop in the first quarter of 2021, with investors appearing to believe that health care would return to the prepandemic status quo and that the necessity of web-based care would wane. By the end of 2020, the CAGR for the US market for the period 2020-2030 had been revised downward from 59.4% to 22.9%, a much more modest growth projection, projecting a US market size of US $140.7 billion by 2030 [5] (see Table 1).

**Table 1:** Expected growth of the US telehealth market.

Year	Actual value (Billions of US $)	Projected value (Billions of US $)
2020	17.9	—^a^
2021	22.0	—
2022	—	27.0
2023	—	33.2
2024	—	40.8
2025	—	50.2
2026	—	61.7
2027	—	75.8
2028	—	93.2
2029	—	114.5
2030	—	140.7

^a^Not applicable.

### Current Telehealth Use

These declines appear to have occurred in spite of evidence that telehealth use remains significantly above prepandemic rates. Indeed, as the dust has settled, concerns that demand for telehealth would completely disappear following widespread vaccination have not been realized. An analysis of data collected from the United States Census Bureau’s Household Pulse survey between April 2021 and August 2022 revealed that around 1 in 4 US adults had reported the use of telehealth services in the preceding 4 weeks [7]. The Centers for Disease Control and Prevention determined in its National Health Interview Survey that 37 percent of US adults had used telehealth services at least once in 2021 [8]. These findings are consistent with the broad satisfaction reported by telehealth users, both patients and clinicians. One survey identified by a 2022 systematic review of patient satisfaction with telehealth during the pandemic found that 91% were satisfied with video consultations, while another noted that 88% considered telehealth consultations more convenient than in-person visits [9]. In a systematic review of literature evaluating physician satisfaction, 89% of the 37 included studies reported moderate or high levels of physician satisfaction [10].

**Figure 1:**
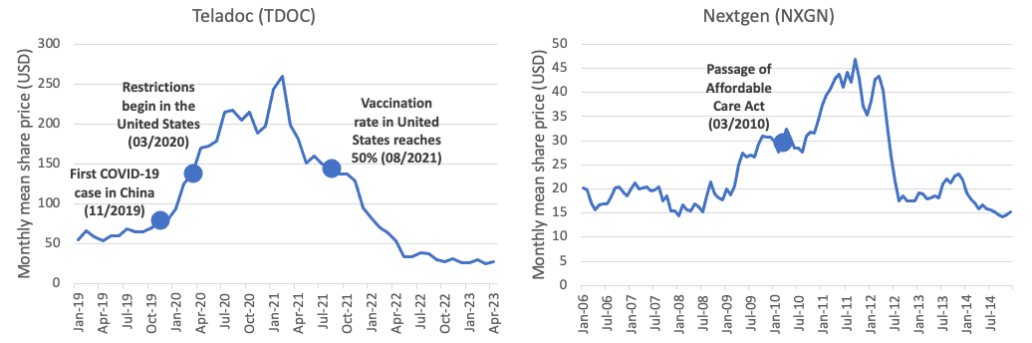
Comparison of Nextgen and Teladoc’s valuation trends.

Why, then, does the financial picture of telehealth seem so gloomy? Closer examination uncovers that a wealth of accessible, pandemic-driven funding [6] resulted in a telehealth market that was quickly overvalued, characterized by higher than reasonable valuations, and is now fragmented as companies compete for industry security. For instance, Teladoc, one of the largest publicly held telemedicine and web-based health care companies, now has a lower valuation than it did prior to the pandemic, with a share price at the time of writing just 9% of its peak in 2021 (from US $293/share to US $26/share) [11] (see Figure 1). Over the last 3 quarters, the company has consistently failed to meet its earnings targets, and maintaining its profitability has proven challenging [12,13].

### Examples From Electronic Health Record Adoption

Telehealth is not the first industry to have experienced inflated valuations followed by a rebalancing. The Affordable Care Act’s (ACA) Health Information Technology for Economic and Clinical Health provisions used strong fiscal incentives to encourage health care facilities to implement federally approved electronic health record (EHR) systems. This resulted in rapid growth in the EHR space following the passage of the ACA, but the industry grew too quickly to the point of oversaturation, where the volume of product is greater than demand. Now, however, 96% of hospitals have adopted EHR (vs 12.2% pre-ACA) [14], creating opportunities for faster and more efficient care as well as improved interoperability between departments and institutions. EHR provider Nextgen (NXGN) experienced a similar valuation trend to that of Teladoc. Shortly after implementation of the ACA, NXGN’s valuation rose to record highs. They then experienced a sharp drop in valuation followed by a rebalance higher than their original valuation before the spike [14] (see Figure 1). Reflecting on EHR valuations provides investors with a potential model for the valuation patterns of a novel technology that experiences rapid growth. Though there are differences between the utility of EHR technologies and telehealth platforms, this model could provide a reasonable level of confidence that telehealth fluctuations are not indicative of a permanent decline.

## Barriers to Success

Companies in the telehealth industry face several key challenges given the unstable and crowded nature of the market. The primary barriers that we will discuss include the competitive intensity of the market, cost drivers, and regulatory risks resulting in barriers to access.

### Competitive Intensity

Given the growth of the market during the pandemic, there are a large number of competitors vying for similar customers. Many telehealth companies went public after the beginning of the pandemic (eg, Talkspace: June 2021, GoodRx: September 2020, and Amwell: September 2020) resulting in the loss of a “first-mover” advantage and an entrance into a saturated market. These companies may be pushed out or acquired by stronger competitors as reflected by their relatively subpar current financial performances compared to their valuation midpandemic.

### Cost Drivers

These same companies also need to manage cost drivers, or factors triggering a change in the price of their product, such as labor and reimbursement. Many of the platforms directly employing clinicians, including Amwell and Teladoc, need to quickly adjust pricing to meet the market. As it is possible that their labor (ie, clinicians) will migrate to the most advantageous telehealth platform, they have less flexibility to offer lower prices and thus struggle with low use. Teladoc gave insight into these struggles in their 10-K statement, the report detailing their financial performance in the preceding year: to retain some contracts, they began offering per-member-per-month, per-enrollee-per-month, and per-subscriber plans, most likely to maximize use from customers [15]. Reducing costs and enhancing efficiency will be key motivators for the longevity and support of telehealth.

Coverage is already being reduced by some private payers, driving up costs to consumers. Currently, economies of scale, or reductions in costs due to high use, are difficult to achieve and may not sufficiently motivate companies to offer web-based health options to their employees, especially on a per-patient pay scale. However, some direct-to-consumer companies claim that by replacing in-person visits with telehealth, the cost incurred by the patient can reach as little as US $40, not to speak of the intangible cost savings of reduced travel time to and from appointments [16]. Still, there are potential hidden costs if the telehealth visit is more likely to result in subsequent in-person appointments or increased testing or prescriptions. Additionally, the convenience of direct-to-consumer telehealth may drive many patients to seek care for an illness who would not have sought care if telehealth had not been available [17].

### Regulatory Risks and Barriers to Access

Beyond competitive and cost risks, shifting regulatory landscapes and variable reimbursement also need to be considered. Regulatory risks are a key factor as restrictions appear to be tightening as governments scale back their pandemic regulations. For example, a waiver for public health emergencies allowed telehealth to be provided for Medicare beneficiaries outside of rural areas and from home rather than from a provider’s office, but the Biden Administration signaled its intention to end the public health emergency on May 11, 2023, and has only guaranteed that the waiver of geographic restrictions on reimbursement of nonbehavioral telehealth care will continue through the end of 2024 [18,19]. Further, telehealth will no longer be classified as an excepted benefit under Medicare once the emergency has expired [16].

But some regulatory changes that facilitated the expanded use of telehealth have been made permanent, such as the Centers for Medicare and Medicaid Services’ expansion of reimbursable telehealth codes for the 2021 physician fee schedule and the elimination of geographic restrictions on reimbursement of behavioral telehealth services [18,19]. Other strategies to increase use include eliminating language barriers and connectivity challenges. This would extend to underserved populations without Wi-Fi or video connection, or patients with a general inability to manage the technology. When a group of 1040 clinicians was surveyed, 73% (759) “felt their patients could navigate the technology without help,” however, 40% (416) reported that “technical issues hindered the start or continuation of the video visit” [20]. A logistic regression of physician responses to a survey on their satisfaction with telehealth identified a strong positive correlation between audio or image quality and provider satisfaction [21].

## Strategies for Sustained Use and Market Penetration

### Identifying the Advantages of Telehealth

In light of the challenges present, strategies for success in the telehealth space moving forward must capitalize on the advantages telehealth provides over traditional care, and consider these advantages in both the delivery models they develop and patients they target. For instance, advantages of telehealth, such as the ability to see a provider outside of traditional working hours, or the opportunity to speak to a niche specialist that may not be local to the patient, differentiate telehealth from in-person care.

Beyond these advantages, health providers must also consider the substance of the visit. Visits for conditions that will include discussion of symptoms versus physical examinations can more sustainably become web-based. Similarly, if no laboratory tests are required, or if laboratory tests can be obtained by the patient for instance through Continuous Glucose Monitoring, a web-based visit can be sufficient.

These advantages and factors are crucial when evaluating the strategies telehealth companies use. These strategies include both the method of expansion—being a web-based first provider or an in-person first provider, as well as the medical specialties a company chooses to offer. We believe consideration of both of these factors is crucial to obtain longstanding success in this space.

### Web-Based First Versus In-Person First Expansion

#### Web-Based First

In its efforts to overcome some of the challenges discussed above, the example of Teladoc again proves illustrative. Recall that Teladoc’s stock market success in the early days of the pandemic did not endure as the interruptions to in-person life precipitated by the pandemic lessened. Yet, over the same period that its valuation decreased, its revenue has grown at rates in the double digits, driven by a sustained increase in memberships and visits [12,13]. Teladoc’s disappointing results were attributed in part to its acquisition of Livongo, a health technology firm that develops remote management technologies for chronic illnesses such as diabetes mellitus [13]. Like many of its telehealth peers at the time of its acquisition, Livongo’s valuation was inflated, and as the market rationalized, or adjusted downward for these high valuations, Teladoc was forced to post a goodwill impairment charge (asset write-off) of US $9.6 billion, triggering investor panic [13]. However, since the purchase, Teladoc has begun closing the gap between its revenue and its profits; while costly to its balance sheet, Livongo has enabled Teladoc to retain members by facilitating its expansion into chronic care [13]. Indeed, Teladoc reported just a 7% overall increase in memberships between 2021 and 2022, but a 16% increase in users of its chronic care program [22].

#### In-Person First

While Teladoc, which entered the market as a platform for telehealth consultations, has sought to secure its place in the market by expanding its suite of web-based offerings to include chronic care, companies with an existing footprint in traditional in-person care are now expanding their offerings to include telehealth services. Earlier this year, CVS Health—parent of CVS Pharmacy, the largest pharmacy chain in the United States by prescription revenue, and Aetna, the sixth largest private health insurer by direct written premiums [23,24]—launched a new web-based primary care offering, which places round-the-clock on-demand care at the disposal of all Aetna commercial members [25]. This followed its acquisition last September of Signify Health, a health technology platform with 10,000 clinicians on staff providing in-home and web-based care, for US $8 billion [26].

One observes, therefore, from 1 corner a move by “telehealth-first” companies to expand their business with a wider range of products more reflective of a traditional provider, akin to developing web-based “departments” for chronic care, primary care, and others, much like a traditional hospital may have different wings and buildings. From the other corner is a move by traditional companies to bring telehealth offerings into their portfolio, both by acquisitions and the in-house launch of new services. As shown in this example, the approach of the telehealth-first vendor is horizontal growth, when a company tries to spread its existing product into new markets, in this case, new specialties. The larger health care industry incumbents, such as CVS, pursue vertical growth, or expansion to new product areas, in this case, telehealth. In both cases, there is confidence in the promise of telehealth, with the result that the telehealth industry of the coming years is likely to consolidate into a smaller range of firms offering a much wider range of services.

### Importance of Medical Specialty

Certain medical specialties also appear better suited to maintaining their hold on the market than others. Telling was an analysis of patient visits at the Duke University Health System between December 2019 and October 2020 which tracked visit volume by type (in-person, video, or telephone) and specialty. The research found that telehealth visits in orthopedics, dermatology, and cardiology increased noticeably in the first months of the pandemic before returning to levels at or slightly above those measured prepandemic by the end of the study period, while consultations in psychiatry and endocrinology moved from in-person to telehealth en masse in the final week of March 2020 and remained high even by the final week of September 2020 [27]. Meanwhile, a time-based study at Penn Medicine Department of Orthopedics found a 0.8% (US $183,456) negative impact on their revenue through the use of telehealth [28]. Thus, while some specialties, such as orthopedics, may not be suitable for telehealth in its current form, there are substantial opportunities in others.

Nearly 75% of health care expenditures are on chronic disease, which is well suited for remote care and monitoring; recall here Teladoc’s recent bet on Livongo [16]. Visits for behavioral health or psychiatry and substance use treatment remain the highest in terms of telehealth use [29], but there have also been promising developments in the use of telehealth to manage other chronic conditions, including diabetes, epilepsy, and rare cancers.

Within endocrinology, uncomplicated diabetes and obesity are well suited to remote management through web-based visits and cloud-based monitoring. Studies evaluating the transition to telehealth during the COVID-19 pandemic have even reported improvements over face-to-face care. At 1 center, the implementation of telephone-based consultations and remote monitoring of glucose levels in patients with type 1 diabetes delivered statistically significant improvements in glycemic control, with mean blood glucose and time spent in hyperglycemia decreasing by 3% and 2.9%, respectively, following the transition [30]. At another single center that switched to telehealth for glycemic control in its diabetic patients, a retrospective review revealed significantly higher attendance at telehealth consultations versus face-to-face appointments [31]. The Cleveland Clinic reported that going digital allowed staff to contact all patients aged 60 years and older with a hemoglobin HbA_1c_>9% seen within the past year and offer them a telehealth appointment to discuss their diabetes management. Furthermore, when they implemented web-based medical appointments for obesity, weight loss at 6 months was found to be similar to that achieved face-to-face. Overall, 90% of the Cleveland Clinic’s endocrinology and metabolism institute patients are now seen digitally [32].

The management of epilepsy has also shown promise as an area into which telehealth might expand. A cohort study comparing outcomes in adults with epilepsy receiving care either remotely or in-person observed no significant difference in the number of seizures, hospitalizations, or emergency room visits between the 2 groups [33], suggesting that telehealth is not deleterious to the management of epilepsy. However, telehealth management is advantaged by the ability of clinicians to deliver care in the home, school, or workplace [34]. This advantage is particularly beneficial to pediatric populations, as travel to and from face-to-face consultations can disrupt both the education of the child and the work or domestic responsibilities of their parent or guardian [35]. A prospective comparison study between face-to-face clinic visits versus telehealth visits among patients with epilepsy and their families found that, while many still indicated a preference for in-person care, satisfaction levels were equal across both visit types, and patients were less likely to cancel telehealth appointments than face-to-face consultations, which may improve long-term management of the condition [36].

The use of supplementary telehealth interventions in oncology has also been associated with significant quality-of-life gains for patients across all cancer types [37], and a comparison between clinical outcomes of patients receiving care by telehealth versus face-to-face found no significant difference in time to staging imaging, time to therapy initiation, or all-cause emergency department presentations [38]. While the frequent necessity of physical examinations may be a challenge to wider adoption [39], the calculus changes in patients battling rarer cancers, such as multiple myeloma; with fewer available specialists, it is not uncommon for such patients to travel long distances in order to access appropriate care [40]. A survey of patients and clinicians at the Royal Marsden Hospital’s Sarcoma Unit on their experiences with telehealth during the pandemic found large majorities in support of continued telehealth use postpandemic. Patients cited reduced cost and travel time as particular benefits of remote care, with the average participant living greater than 1.5 hours from the Unit by car or transit. Meanwhile, clinicians were satisfied with the efficiency of telehealth, did not report increased workload, and almost all believed telehealth was practice-changing. Despite the concern that physical examinations may impede the transition to telehealth, clinicians did not find that the decrease in these visits due to the pandemic often affected care provision [41].

For these indications, the transition to telehealth has been demonstrably beneficial to patients without sacrificing quality of care. This is particularly the case in chronic conditions, which require long-term management and therefore impose significant demands on the time and resources of patients and clinicians alike. Leveraging telehealth as a means to expand access to care and improve the quality of life among those affected by such conditions could prove revolutionary. Further, the observed decreases in travel time, costs, and cancelations with telehealth consultations may increase patient adherence to their therapies and in turn, lead to superior clinical outcomes over face-to-face care.

### Conclusions

Low barriers to entry coupled with pandemic-driven market opportunity allowed telehealth to achieve impressive growth over the past 3 years. However, as pandemic pressures on in-person health care in the United States appear to be lifting, the market has faced increased challenges. Competitive intensity, pricing wars, and barriers to accessing care all serve as roadblocks. To overcome competitors and gain a market advantage, companies need to be aware of the driving factors motivating patients and clinicians including cost, convenience, and efficacy. Success will come only from changing the actual models of care, not simply moving the same care over the internet, and developing integrative platforms in which multiple forms of telehealth technologies are leveraged to deliver an entirely new and more comprehensive product. In the case of health care giants, this is likely to emerge from vertical expansion—the addition of telehealth services to their existing in-person care portfolio—while for newer telehealth businesses expansion must be horizontal, adding new types of service for new indications to their existing web-based platforms.

Moreover, it is not merely the quantity of acquisitions and expansions but their strategic value which will ultimately bridge the gap between ostensible investor pessimism and the demonstrated promise of the telehealth market. For instance, the decision by some telehealth companies to target specific types of care, such as the treatment of chronic illnesses and psychiatric disorders requiring long-term management, is likely to be driven by the suitability of those particular indications to management via telehealth. Slow but continued growth can be expected as companies continue to better understand their market and target customers.

From the investment side, the drop in valuations across the telehealth space may seem inescapable. However, the overcapacity and fragmented market seems to be rebalancing, and from this process could emerge telehealth giants who, by strategic expansion strategies suited to their position in the health care industry as a whole, are able to adapt to the changing landscape. With potential new pandemics looming on the horizon, telehealth is a sector that could realize massive gains, as well as provide increased access to care for patients. This is an industry that deserves continued attention.

## Abbreviations

ACA: Affordable Care Act
CAGR: compound annual growth rate
EHR: electronic health record
NXGN: Nextgen
